# The Crosstalk between Microbiome and Immune Response in Gastric Cancer

**DOI:** 10.3390/ijms21186586

**Published:** 2020-09-09

**Authors:** Rihab Nasr, Ali Shamseddine, Deborah Mukherji, Farah Nassar, Sally Temraz

**Affiliations:** 1 Department of Anatomy, Cell Biology and Physiology, American University of Beirut Medical Center, Riad El Solh, Beirut 1107, Lebanon; rn03@aub.edu.lb; 2 Department of Internal Medicine, Hematology/Oncology Division, American University of Beirut Medical Center, Riad El Solh, Beirut 1107, Lebanon; as04@aub.edu.lb (A.S.); dm25@aub.edu.lb (D.M.); fn17@aub.edu.lb (F.N.)

**Keywords:** gut microbiome, *H. pylori*, lactic acid bacteria, immune checkpoint inhibitors, immunotherapy, immune response, gastric cancer, PD-L1, CTLA-4

## Abstract

Gastric cancer is the end result of a complex interplay between host genetics, environmental factors, and microbial factors. The link between gut microbiome and gastric cancer has been attributed to persistent activation of the host’s immune system by gut microbiota. The end result of this dysregulated interaction between host epithelium and microbes is a state of chronic inflammation. Gut bacteria can promote anti-tumor immune responses through several mechanisms. These include triggering T-cell responses to bacterial antigens that can cross-react with tumor antigens or cause tumor-specific antigen recognition; engagement of pattern recognition receptors that mediate pro-immune or anti-inflammatory effects or via small metabolites that mediate systemic effects on the host. Here we review the role of the gut microbiome including *H. pylori* and non-*H. pylori* gastric bacteria, the immune response, and immunotherapy using checkpoint inhibitors. We also review the evidence for cross talk between the gut microbiome and immune response in gastric cancer.

## 1. Introduction

The gastrointestinal tract represents the largest microbial ecosystem in the human body and the gut microbiome is defined as the total genomic content of the complex microbial communities (the microbiota including bacteria, viruses, and archaea) and elements of the host such as the host epithelium, immune system, and products of both the microbes and host including metabolites. In normal “symbiosis”, the role of the gut microbiome is to support the host’s mucosal immune response, energy metabolism, pathogen elimination, and prevent cancer development [1]. When normal “symbiosis” switches to “dysbiosis” which represents a change in the numbers of microbes or a change in the diversity of the microbiota, the normal cohabitants of our gut transform into “pathobionts” and, in accordance with dietary carcinogens, smoking, alcohol, and other environmental factors, they promote carcinogenesis.

Gastric cancer is the fifth leading cancer type and has been identified as one of the main causes of cancer-related deaths worldwide [2]. Gastric cancer is the end result of a complex interplay between host genetics [3,4], environmental factors (e.g., smoking, alcohol, high salt and meat intake, low fruit/vegetable intake), and microbial factors [5] (i.e., *Helicobacter pylori* infection and the gut microbiota). The link between gut microbiome and gastric cancer has been attributed to persistent activation of the host’s immune system by gut microbiota resulting in dysregulated interaction between host epithelium and microbes and a state of chronic inflammation. In this review, we will examine the role of microbiome in modulating immune response in gastric cancer.

## 2. Gut Microbiome and Gastric Cancer

The human gut hosts a diverse community of bacteria referred to as the gut microbiome. Human gut-associated microbiota are dominated by four main phyla: Firmicutes, Bacteroidetes, Actinobacteria, and Proteobacteria. Firmicutes consisting of Clostridium, Ruminococcus, Eubacterium, Dorea, Peptostreptococcus, and *Lactobacillus-L* are the most frequently occurring, representing around 30.6–83%. Next are Bacteroidetes, consisting of Bacteroides and representing 8 to 48%, followed by Actinobacteria, consisting of Bifidobacterium, representing 0.7 to 16.7%, and finally Proteobacteria, consisting of Enterobacteriaceae and representing 0.1 to 26.6% [6]. Studies have shown that changes in bacterial composition and a decrease in diversity of the microbiome disrupt its physiological interaction with the gut immune system, leading to chronic inflammation and cancer.

### 2.1. Mechanism by Which Microbiota Induce Tumorigenesis

The gut microbiome is involved in major steps of carcinogenesis, including tumor-promoting inflammation [7], altered immune response [8], tumor growth [9], angiogenesis [10], pro-carcinogenic metabolite production [11], DNA damage, and induction of genomic instability [12].

The immune system contains an immunologic archive based on pattern recognition receptors (PPR) which are able to distinguish potentially pathogenic microorganisms from harmless commensals. PPR primarily recognizes surface molecules derived from microbes, especially bacterial lipopolysccarides, lipoproteins, prokaryotic DNA, and foreign nucleic acids, so-called microorganism-associated molecular patterns (MAMPs) or pathogen-associated molecular patterns (PAMPs). Toll-like receptors (TLR) belong to a major class of PRR expressed on membranes of macrophages and dendritic cells. Another group of PRR is NOD-like receptors (NLRs). The NLRs relate to a large family of cytosolic innate receptors involved in detection of intracellular pathogens and endogenous byproducts of tissue injury.

**Figure 1:**
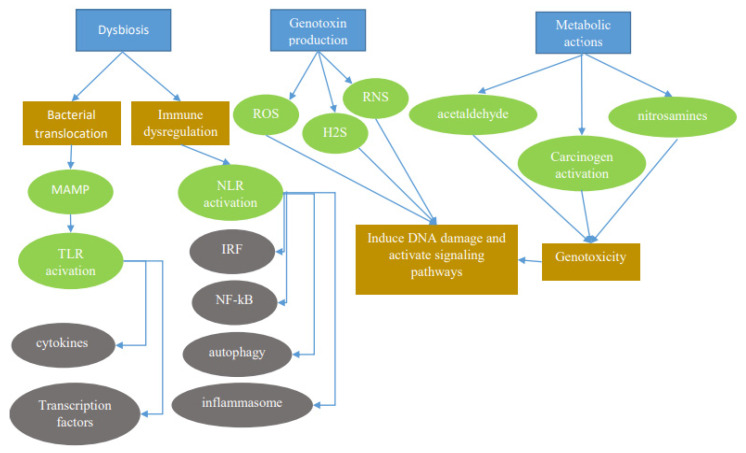
The microbiota promotes carcinogenesis through different mechanisms (blue rectangles). Dysbiosis can induce carcinogenesis through bacterial translocation and immune dysregulation. Through bacterial translocation, microorganism-associated molecular patterns (MAMPs) activate Toll-like receptors (TLRs), which in turn activate cytokines and transcription factors. Through immune dysregulation, nod-like receptors (NLRs) activate multiple signal pathways to induce the formation of inflammasomes and/or activate nuclear factor κB (NF-κB), stress kinases, interferon regulatory factors (IRFs), inflammatory caspases, and autophagy Genotoxins such as reactive oxygen species (ROS), reactive nitrogen species (RNS), and hydrogen sulfide (H2S) released by certain bacteria can have detrimental effects. Also, metabolic actions of bacteria activating toxins such as acetaldehydes and nitrosamines can also result in a genotoxic effect leading to carcinogenesis.

Dysbiosis favors invasion and growth of pathogenic species and disrupt homeostasis of the immune system and mucosal barrier. The subsequent inflammatory process results in increased permeability, allowing gut microbes to drive a continuous state of inflammation, thereby activating TLR and NLR signaling (Figure 1). TLR signaling is transduced via adaptor proteins such as myeloid differentiation primary response-88 (MyD88) and TIR-domain-containing adapter-inducing interferon-β (TRIF). MyD88 and TRIF signaling lead to expression of cytokines such as tumor necrosis factor- α (TNF-α), interleukin-1 beta (IL-1β), interleukin-6 (IL-6), interferon gamma-induced protein 10 (IP-10), and interferon-γ (IFN-γ) through the activation of transcriptional factors nuclear factor κB (NF-κB), activator protein 1 (AP-1), and interferon regulatory factor 3 (IRF-3) [13]. NLR activation triggers structural rearrangement of the receptor to conduct signal spread activating multiple signal pathways to induce the formation of inflammasomes and/or activate NF-κB, stress kinases, IRFs, inflammatory caspases, and autophagy [14].

Microbiota can produce pro-carcinogenic metabolites. A good example of such a metabolite is butyrate, which is produced by bacterial species through the anaerobic fermentation of carbohydrates and provides an important energy source for host cells such as colonocytes. While butyrate has been shown in a range of studies to have beneficial anti-cancer effects, in the right genetic background, butyrate promotes carcinogenesis through the increased proliferation of aberrant epithelial cells [15].

Microbiota may also cause genotoxic effects which can damage host DNA and activate signaling cascades (Figure 1). The resulting chromosomal aberrations and translocation of microbial processes result in the activation of interleukin (IL)-23-producing myeloid cells, which in turn promote tumor growth [16]. Similarly, reactive oxygen species (ROS) and reactive nitrogen species generated by inflammatory cells and hydrogen sulfide (H2S) released by bacterial microbiota may also be genotoxic [17]. Metabolic actions of the microbiome consisting of the activation of other genotoxins such as acetaldehyde, dietary nitrosamines, and other carcinogens constitute another pathway by which the microbiome exerts its effect on the host [17].

### 2.2. Microbiota Implicated in Gastric Cancer

#### 2.2.1. Helicobacter Pylori (*H. pylori*)

*H. pylori* is a Gram-negative member of the Epsilonproteobacteria class which has been classified as Class I carcinogen by the World Health Organization [18]. *H. pylori* infection changes the composition of gastric microbiota by increasing gastric pH and creating special niches for bacterial colonization. Prolonged *H. pylori* infection prompts a chronic state of inflammation leading to the sequential development of gastritis, gastric ulcer, atrophy, and gastric cancer. Around 50% of the world’s population has *H. pylori* infection, but only 1 to 3% of these progress to gastric cancer and 0.1% develop mucosa-associated lymphoid tissue (MALT) lymphoma [17].

The mechanism by which *H. pylori* promotes carcinogenic processes is through direct damage to host DNA by converting nitrogen compounds in gastric fluid to potentially carcinogenic N-nitroso compounds (NOCs), reactive nitrogen intermediates, dysregulation of DNA transcription factors such as Caudal type Homebox 2 (Cdx2), and establishment of an inflammatory milieu at the gastric mucosa [19]. A defect in DNA repair is associated with *H. pylori* gastritis leading to increased mutagenesis in *H. pylori*-infected mucosa. The direct influence of *H. pylori* on carcinogenesis is mediated through two virulence factors: cytotoxin-associated gene A (CagA) and vacuolating cytotoxin A (VacA) [20]. CagA is a strain-specific protein that is translocated into the host cell by the type IV secretion system and acts as a classic oncogene key leading to chronic gastritis and ulceration, MALT lymphoma, and gastric cancer in humans. CagA inhibits the apoptotic pathway of epithelial cells and caused morphological aberrations, including cell scattering and elongation, and loss of cell polarity [21]. VacA is a high molecular-weight multimeric pore-forming protein that is found in all *H. pylori* strains. The persistence of VacA in the human stomach is facilitated through pore formation in the epithelial membrane which results in the subsequent egress of urea, thus enabling *H. pylori* to catalyze urea hydrolysis as a means of protection against gastric acidity and the suppression of macrophages and T-cells [22]. Furthermore, adherence of *H. pylori* to gastric epithelial cells results in an inflammatory immune response which involves gene alterations in both the adaptive and the innate immune system such as interleukins (IL1β, IL8), transcription factors (CDX2, RUNX3, TLR1), and DNA repair enzymes [23,24].

*H. pylori*-induced gastritis can be antral- or corpus-predominant [22]. In antral-predominant gastritis, *H. pylori*-mediated increased gastrin secretion leads to more gastric acid production, which makes patients more vulnerable to duodenal ulcers but protects them against gastric cancer. On the other hand, in corpus-predominant gastritis, *H. pylori* suppresses acid production through inflammatory mediators, which leads to the progressive loss of gastric glands and eventually atrophic gastritis [22].

*H. pylori* eradication therapy has been shown to be effective in preventing gastric cancer mainly through halting the phenomenon of increased nitrosating bacteria [25]. In intestinal type gastric cancer, the Correa Cascade illustrates the stages of gastric carcinogenesis from precancerous lesions—superficial gastritis, chronic atrophic gastritis, intestinal metaplasia, and dysplasia—to invasive neoplasia [26]. Whether eradication of *H. pylori* has the potential to stop or even reverse this process and prevent carcinogenesis at any stage of this cascade, or if there is a point of no return, have been studied. A randomized controlled trial enrolling 1630 *H. pylori* carriers with a 7.5-year follow-up did not find any benefit of *H. pylori* eradication [27]. However, further subgroup analysis revealed that *H. pylori* eradication in patients without precancerous lesions (such as gastric atrophy, intestinal metaplasia, and gastric dysplasia) significantly decreased the incidence of gastric cancer, indicating that after a certain time point, *H. pylori* has a limited effect on carcinogenesis [27]. Furthermore, in a recent prospective cohort of 1755 patients with dyspepsia who underwent Operative link on gastritis assessment (OLGA) staging and assessment of *H. pylori* infection revealed that *H. pylori* eradication in subjects with advanced stages (III-IV) did not abolish the risk for neoplastic progression [28]. Recently, even in patients being treated for early gastric cancer, *H. pylori* eradication was shown to still be effective in a subset of patients by minimizing the risk of metachronous gastric cancer [25,29]. Thus, *H. pylori* chronic infection plays an important role in the early stages of gastric cancer; however, its colonization in atrophy and intestinal metaplasia is scarce, leading to the hypothesis that other bacteria within the gastric microbiome are also involved in gastric cancer development.

#### 2.2.2. Non-*H. pylori* Gastric Bacteria

With the application of metagenomics and high throughput sequencing technology in microbiology, the stomach, which was once thought to be a sterile organ, was found to harbor other acid-resistant bacteria besides *H. pylori*. The gastric bacterial community was found to be dominated by five major phyla: *Proteobacteria*, *Firmicutes*, *Bacteroidetes*, *Actinobacteria* and *Fusobacteria* [30]. Table 1 reveals the major studies in which non-*H. pylori* have been implicated in some stages of gastric cancer development. In summary, data up till now reveal that the bacterial genera most consistently reported to be enriched in patients with gastric adenocarcinoma include Lactobacillus, Streptococcus, Veillonella, Prevotella, Fusobacterium, Lachnospiraceae, Leptotrichia, and Clostridium whereas those most consistently reported in intestinal metaplasia include Streptococcus, Prevotella, Fusobacterium, and Leptotrichia (Table 1).

Most of the aforementioned non-*H. pylori* gastric bacteria implicated in gastric cancer are lactic acid bacteria (LAB) which include Streptococcus, Lactobacillus, Bifidobacterium, and Lactococcus. There are several means by which LAB can influence gastric cancer development. First, LAB can increase N-nitroso compounds which have been shown to promote mutagenesis, angiogenesis, proto oncogene expression and inhibit apoptosis [46,47] and increase ROS which induce DNA damage [48]. Second, LAB can increase epithelial mesenchymal transition by inducing multipotency and contributing to tumor progression [49,50]. Third, LAB can promote colonization by non-*H. pylori* carcinogenic pathobionts by inducing immune tolerance [51]. And finally, LAB can augment production of exogenous lactate which is involved in several hallmarks of cancer and regulates the expression of important key players in cancer development [52].

Lactate, which is normally produced in the average human in the order of 0.8 mmol/kg body weight, is found in glycolytic tumors at concentrations of over ten times this value ranging between 10 to 12.9 mmol/kg. This increased lactate concentration can serve as a fuel source for oxidative cancer cells, upregulating monocarboxylate transporter 1 (MCT1) and consecutively contributing to cell migration [53,54]. Lactate can also activate hypoxia-inducible factor-1 (HIF-1) which in turn induces epithelial mesenchymal transition. Through lactate-mediated expression of hydrocarboxylic acid receptor 1 (HCAR1) and MCT4, lactate can contribute to chemoresistance [55] and can promote tumor growth [56,57]. Lactate also mediates M2-like polarization of tumor associated macrophages, which is believed to be tumor supportive [58], and increases the expression of vascular endothelial growth factor and arginase 1 [59] eventually leading to immune escape [60]. And finally, lactate inhibits T and natural killer cells function and survival [61] and increases the amount of myeloid derived suppressor cells which can further suppress natural killer cell cytotoxicity [62].

## 3. Immune Response in Gastric Cancer

Altered proteins produced from mutated genes or viral genes are recognized as tumor antigens by immune cells regardless of their function. Neoantigens can arise from these altered tumor proteins and be presented on tumor cell surface via major histocompatibility complex (MHC). Newly formed antigens on tumor cell surfaces are recognized by the immune system which triggers the immune response Cytotoxic T cells express CD8 and CD3, and have T cell receptors (TCRs) that recognize tumor antigens presented by MHC class I molecules [63]. T cell proliferation and activation in tumor tissues requires stimulation by two stimuli. The first signal involves the binding between neoantigen presented on MHC molecule and TCR. The second signal is co-inhibitory or co-stimulatory and determines whether T cells will be activated or not.

T cells have co-stimulatory receptors that combine with expressed co-stimulatory ligands on the surface of tumor cells to promote T cell activation. T cells must receive co-stimulation via engagement of CD28 on their surface with CD80 or CD86, which are also, respectively, known as B7-1 and B7-2, on the surface of antigen-presenting cells. The signals delivered via CD28 affect crucial events in T cells, such as transcriptional signaling, post-translational protein modifications, cytokine synthesis and epigenetic changes that ultimately affect their phenotype and function. The CD28 ligands, CD80, and CD86 vary in their expression pattern. CD86 is constitutively expressed on antigen-presenting cells and is upregulated quickly during immune responses, whereas CD80 is slower in its upregulation [64].

In contrast to the co-stimulatory receptors described, tumor cells produce several co-inhibitory receptors, including programmed cell death protein-1 (PD-1), lymphocyte-activation gene 3 (LAG 3), T cell immunoglobulin and mucin domain containing-3 (TIM-3). Binding between co-inhibitory receptors and their ligands induces T cell inactivation. PD-1 represents a co-inhibitory receptor that is found on the surface of several types of cells, such as activated T cells, T regulatory cells (Tregs) and monocytes. It has two ligands, PD-L1 and PD-L2. PD-L1 is expressed on both immune and tumor cells, while PD-L2 is mostly expressed on antigen-presenting cells. Tumor-infiltrating lymphocytes (TILS) release IFN-γ and induce expression of PD-L1 in surrounding tumor cells, stromal cells, and blood cells [65]. PD-L1 that is expressed on tumor cells binds to PD-1 on activated T cells that reach the tumor and generates a suppression signal for the activation of T cells, which become unable to destroy tumor cells, leading to a decrease in both cellular and humoral immune responses [66,67]. The PD-1/PD-L1/2 pathway seems to protect tumor cells from attack by T lymphocytes. Cytotoxic T lymphocyte–associated protein 4 (CTLA-4) is a co-inhibitory molecule exhibited on activated T lymphocytes and Tregs, whose receptor on T cells interacts with its B7-1/B7-2 ligands located on antigen-presenting cells consequently suppressing the T cell stimulatory signal mediated by CD28 [68]. CTLA-4 expression is stimulated only in the context of T cell activation; afterwards, it competes with CD28 to bind to B7 molecules and decrease the immune response. T regulatory cells (Tregs) express CD4, FOXP3 and CD25. Tregs suppress the immune response to self-proteins and the tumor immune response [69]. They inhibit the tumor immune response by producing high-affinity interleukin-2 (IL-2) receptor, CTLA-4, IL-10 and immunosuppressive cytokines such as transforming growth factor β (TGF-β). LAG-3 expression is increased in activated T cells and natural killer cells, and its ligands are MHC class II, LSECtin, and galectin-3 [70]. LAG-3 inhibits T cell proliferation and cytokine production. TIM-3 binds to its ligands, including galectin-9, high mobility group box 1 (HMGB1), and carcinoembryonic antigen cell adhesion molecule 1 and plays a role in immune evasion of tumor cells by inactivating T cells [71]. Blockade of these co-inhibitory signals is the basic strategy for cancer immunotherapy.

### 3.1. Immunotherapy with Checkpoint Inhibitors in Gastric Cancer

**Figure 2:**
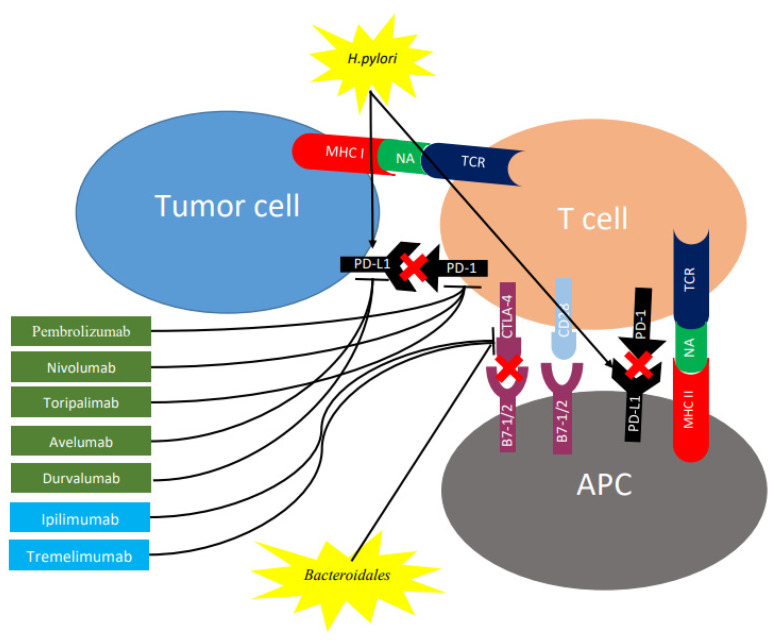
Role of checkpoint inhibitors and gut microbiome on expression of CTLA-4 and PD-1 in regulating different stages of T cell response. T cell activation requires two complementary signals: The interaction between the TCR and peptide-MHC complex must be associated with a second co-stimulatory signal mediated by CD28. Conversely, the binding of CTLA4 to B7-1/2 provides a control signal that suppresses ongoing T cell activation. PD-1 is upregulated on T cells following persistent antigen exposure. When PD1 binds to its ligand, PD-L1 or PD-L2, expressed by tumor cells, the T cell receives an inhibitory signal. Antibodies against CTLA-4 (shown in blue rectangles) or PD-1/PD-L1 (shown in green rectangles) can activate T cells. *H. pylori* increases gastric epithelial expression of PD-L1 while bacteroides block CTLA-4 expression. CTLA-4, cytotoxic T lymphocyte antigen 4; PD-1, programmed cell death protein 1; TCR, T cell receptor; MHC, major histocompatibility complex; PD-L1, programmed death ligand 1; APC, antigen-presenting cell; NA, neoantigen.

Immune checkpoints represent inhibitory pathways that are critical for maintaining self-tolerance and physiological homeostasis by controlling the intensity of physiological immune responses to prevent tissue injury, particularly when the immune system is fighting an infection. Additionally, they may also allow immune escape of cancer cells. Immune checkpoint molecules, such as CTLA-4 and PD-1/PD-L1, are involved in the inhibition of T cell activation via different pathways (Figure 2).

By inhibiting the interaction between CTLA-4 and B7 on antigen-presenting cells through the use of an antiCTLA-4 antibody (Figure 2), T cell activation and proliferation is promoted, along with a decrease in immunosuppressive Treg cells among TILs [72]. Conversely, inducing antibody-mediate blockage of the PD-1/PD-L1 pathway, followed by the inhibition of this checkpoint (Figure 2), treatment is able to enhance the anticancer immune response of the host [73]. Table 2 shows the main checkpoint inhibitors for CTLA-4 and PD-1/PD-L1, which have been studied in the context of gastric cancer.

### 3.2. Biomarkers for Immunotherapy

Immunotherapy has changed the therapeutic strategy for patients with gastric cancer and has improved overall survival and clinical responses. Unfortunately, the response rate remains low, and the predictive factors that will identify the subgroup of patients who derive the greater benefit of therapy should be determined. Thus, several biomarkers have been evaluated for achievement of clinical benefit in gastric cancer.

#### 3.2.1. Programmed Death Ligand 1

Tumor cells and associated stromal cells can express PD-L1, thereby turning off T-cell activation and allowing uncontrolled tumor cell proliferation. Therefore, PD-L1 expression has been considered to be one of the most promising biomarkers for anti-PD-1 drugs [82]. Saito et al. showed that PD-1 expression on CD4+ and CD8+ T cells from gastric cancer patients was significantly higher than that from normal controls [83]. Moreover, PD-L1 expression was encountered in 42% of gastric cancer tissues, but not in normal gastric mucosa; it is particularly specific for Epstein-Barr virus (EBV) positive and microsatellite instability (MSI)-H subtypes [84]. In KEYNOTE-061, PD-L1 expression has been correlated with a better treatment outcome with pembrolizumab. These data reinforce the utility of PD-L1 expression for selecting patients for treatment with pembrolizumab monotherapy. In the KEYNOTE-061, PD-L1 expression was prospectively assessed on tumor cells and tumor-associated lymphocytes and macrophages using the 22C3 pharmDx assay [85]. PD-L1 expression assessed by this assay can be quantified by the combined positive score (CPS) method, which is the number of PD-L1 staining cells (tumor cells, lymphocytes, and macrophages) divided by the total number of viable tumor cells, multiplied by 100. If the result of the calculation exceeds 100, the maximum score is regarded as CPS 100. A tumor with CPS ≥ 1 score is considered positive for PD-L1 expression. For adequate evaluation, at least 100 viable tumor cells are needed in a stained slide. On the contrary, results from the ATTRACTION-2 study, which assessed the expression of PD-L1 using 28-8 pharmDx assay, showed a significant benefit of nivolumab in all patients [76]. This assay utilized the tumor proportion score (TPS), which is evaluation of membrane staining of PD-L1 expression on tumor cells with a PD-L1 positivity defined as TPS ≥ 1.

#### 3.2.2. Tumor-Infiltrating Lymphocytes (TILs)

TILs comprise the presence of T cells, B cells, and NK cells with specific immunological reactivity against tumor cell [86]. The absence of TILs may contribute to immunotherapy resistance [87]. T cells include cytotoxic lymphocytes (CD8+), helper T cells (CD4+), memory T cells (CD45RO+) and Tregs (FOXP3+). Stromal TILs represent the mononuclear inflammatory cells infiltrating tumor stroma, whereas intratumor TILs are defined as the intraepithelial lymphocytes/mononuclear cells within the tumor. The assessment of TILs as a prognostic biomarker in gastric cancer patients has led to controversial conclusions. Studies have shown that high density of intratumor TILs are associated with better prognosis [88,89]. In one study, increased CD8+ T cells both intra or extra-tumor located have been associated with improved disease free and overall survival [90,91] but was shown to be correlated with poor overall survival and increased expression of PD-L1 in another study [92]. Other studies showed that a high density of intratumor FOXP3+ Treg is correlated with a poor overall survival, whereas an extratumor high density of this cell type leads to an increased overall survival [93,94]. Moreover, a better overall survival was associated with increased intratumor CD3+ T cells [94,95] and CD57 NK [94,96]. Thus, data from the literature suggest that high CD8+, CD3+ and CD57+ TILs and low FOXp3+ Tregs are favorable prognostic factors in gastric neoplasia.

#### 3.2.3. Microsatellite Instability (MSI)

The Cancer Genome Atlas has categorized gastric cancers into the following four molecular subtypes: Epstein–Barr virus (EBV)-positive, MSI-high, genomically stable and chromosomally instable [97]. MSI-high tumors are representative of high mutational burden and account for 22% of the patients with gastric cancer [97]. MSI-H gastric cancers are usually associated with antrum location, female gender, relatively older age, earlier stage and Lauren intestinal type [98]. The presence of deficient MisMatch Repair (dMMR) results in tumor cells accumulating frequent genetic mutations. With high mutational burden, tumor cells produce several neo-antigens that trigger T cell activation and recruitment. As the tumor immune reaction increases, expression of checkpoint molecules in tumor cells and immune cells is upregulated [99]. In a post hoc exploratory analysis of KEYNOTE-061, patients with MSI-high tumors showed a large treatment effect with pembrolizumab irrespective of PD-L1 status [78]. Moreover, in a phase II trial assessing response rate of 61 gastric cancer patients treated with pembrolizumab found a response rate of 85.7% in MSI-high tumors [100]. These results suggest that MSI-high gastric cancer subtype is particularly responsive to anti-PD-1 therapy. The FDA approved pembrolizumab for the treatment of adult and pediatric patients with unresectable or metastatic, MSI-H or dMMR solid tumors that have progressed following prior treatment and who have no satisfactory alternative treatment options.

#### 3.2.4. Epstein-Barr Virus

EBV-positive gastric cancers are characterized by marked intra- or peri-tumoral immune cell infiltration and often exhibits the genomic amplification of the chromosome 9 locus containing genes encoding PD-L1 and PD-L2 [101]. EBV-positive gastric cancers have several distinct clinicopathologic characteristics which include: abundant TILs, male predominance, relatively young age, earlier stage and favorable prognosis [97]. The incidence of EBV-positive gastric cancers varies with country and ethnicity, with a range of 2–20.1% and a worldwide average of nearly 10% [97]. Nearly 50% of EBV-positive gastric cancers showed high expression of PD-L1 [102]. Kim et al. reported in their phase II trial a 100% response rate to pembrolizumab in patients with EBV-positive gastric cancer [100]. Of note, the 6 patients who were EBV-positive achieved a partial response with a median duration of 8.5 months in third-line therapy [100]. This suggests that EBV-positivity in gastric cancer could be a predictive biomarker for response to immune checkpoint blockade.

#### 3.2.5. Tumor-Mutational Burden (TMB)

TMB is a new predictive biomarker for response to immunotherapy. It is a quantitative measure of the total number of somatic nonsynonymous mutations per megabase of genome examined in the DNA of cancer cells [103]. TMB has been shown to be associated with good response to immunotherapy and improved survival [103]. Tumors with higher TMB are hypothesized to be more likely to express neoantigens that can be recognized by the immune system in response to immune checkpoint inhibitors [103]. Clinical trial NCT02915432, which investigated the safety and efficacy of toripalimab in Chinese patients with advanced gastric cancer, demonstrated that patients with high TMB showed significant treatment response and overall survival benefit compared to patients with low TMB [79]. The FDA approved pembrolizumab for the treatment of adult and pediatric patients with unresectable or metastatic solid tumors with tissue TMB-H ≥ 10 mutations/megabase.

#### 3.2.6. ctDNA

Plasma-derived ctDNA sequencing has been shown to reproduce tumor tissue exome sequencing for identifying patients who are likely to respond to pembrolizumab [104]. ctDNA mutational load score was shown to be well correlated with response to pembrolizumab and it appeared to predict progression free survival, at least, as well as the tissue mutational load [100]. These data suggest that in patients unable or unwilling to undergo invasive tissue biopsy, broad ctDNA profiling may suffice to accurately identify potential candidates for pembrolizumab therapy. However, this technique fails to identify patients who are EBV-positive.

## 4. The Crosstalk between Gut Microbiome and Immune Response in Gastric Cancer

The gut microbiome plays an important role in gastric cancer carcinogenesis and likely influences response to immunotherapy. Gut bacteria can promote anti-tumor immune responses through several mechanisms, including triggering T-cell responses to bacterial antigens that can cross-react with tumor antigens or cause tumor-specific antigen recognition through engagement of pattern recognition receptors that mediate pro-immune or anti-inflammatory effects or via small metabolites that mediate systemic effects on the host [105].

Peptide or lipid structures from bacteria can activate a range of distinct T cell receptors, thus selecting a surge of T lymphocytes that might be expanded and enter the circulation. Das et al. reported that *H. pylori* increased gastric epithelial expression of PD-L1 and that gastric epithelial cells exposed to *H. pylori* inhibited the proliferation of CD4+ T cells isolated from blood and the inhibitory effect can be blocked using anti PD-L1 antibodies [106]. Also, Wu et al. reported an increase in PD-L1 expression in gastric biopsies of individuals infected with *H. pylori* and co-culture of *H. pylori* infected primary gastric epithelial cells with T cells isolated from blood induced T cell apoptosis [107]. These results suggest that *H. pylori* infection may cause the non-specific inhibition of circulating T cells, including tumor-specific T cells (Figure 2). Recently, Liu et al. observed that PD-L1 expression in the tumors of gastric cancer patients were significantly associated with *H. pylori* status, with the greater proportion of PD-L1 CPS ≥ 1 tumors reported among *H. pylori*-positive as compared to *H. pylori*-negative tumors. There was no association between *H. pylori* and EBV infection in this study, suggesting that cases with *H. pylori* infection are also potential candidates for anti-PD-1 therapy [108].

In terms of effects on pattern recognition receptors, Vétizou et al. found that the antitumor effects of CTLA-4 blockade depend on distinct Bacteroides species (Figure 2). In mice and patients, T cell responses specific for *B. thetaiotaomicron* or *B. fragilis* were associated with the efficacy of CTLA-4 blockade through induction of Il-12-dependent Th1 anti-tumor responses. Tumors in antibiotic-treated or germ-free mice did not respond to CTLA-4 blockade. This defect was overcome by gavage with *B. fragilis*, by immunization with *B. fragilis* polysaccharides, or by adoptive transfer of *B. fragilis*-specific T cells [109]. In the presence of *Bifidobacteria*, type I interferon (IFN)—related immune genes are up-regulated in antigen-presenting cells of secondary lymphoid organs [110]. In patients with advanced cancer, antibiotics inhibit the clinical benefit of immune checkpoint inhibitors. Fecal microbiota transplantation (FMT) from cancer patients, who responded to immune checkpoint inhibitors, into sterile mice enhanced the antitumor effects of PD-1 blockade, whereas FMT from non-responders did not [111].

The gut microbiome has a major impact on host metabolism through generation of small peptides which influence host immune-metabolism. For instance, polyamines such as spermidine and Vitamin B6 generated in the gut, can stimulate autophagy at distant sites of the body, eliciting anticancer immune responses in the context of chemotherapy [112]. Also, short-chain fatty acids produced by gut bacteria are sensed by a variety of cell types, including regulatory T cells expressing the G protein-coupled receptors GPR41 or GPR43 [113]. Dipeptide aldehydes derived from bacteria mediate cathepsin L inhibition, which may enable gut mutualists to stably occupy a niche in the phagolysosome and interfere with antigen presentation of epithelial or immune cells [113]. It is thus anticipated that these and other metabolites may influence the host immune system.

## 5. Conclusions

Recent studies have focused on the gut microbiome as a key player precipitating tumorigenesis and modifying response to treatment. Pre-clinical and human studies have provided evidence on the role of microbiota, specifically bacteria in cancer development and recently response to immunotherapy. Due to the complex and dynamic nature of the human gastrointestinal microbiota, it is considered to be a metabolically active organ and the complex nature of it evidently regulates gastrointestinal homeostasis by interacting with immune cells and influencing response to immunotherapy. One of the striking findings that distinguishes cancer patient responders from non-responders to PD-1 blockade immunotherapy is the ratio of putatively favorable to unfavorable bacteria [114]. Thus, it is conceivable that some commensal organisms have a negative impact on immunotherapy efficacy, while others have a positive one. Strategies aimed at specifically eliminating unfavorable bacteria while providing immune-potentiating effects should be further pursued.

## Figures and Tables

**Table 1:** Studies implicating other non-*H. pylori* bacteria in gastric cancer development.

Study	Study Sample (N)	Methods	Dominant Genera Other Than *H. pylori*
Dicksved et al., 2009 [31]	GAC (10) FD (5)	Terminal RFLP with 16S rRNA sequencing	No significant differences in microbiota composition between GAC and control group. Enriched genera in GAC: Streptococcus, Lactobacillus, Veillonella and Prevotella
Aviles-Jimenez et al., 2014 [32]	GAC (5) IM (5) NAG (5)	Microarray G3 PhyloChi	Gradual change in the gastric microbiota profile from NAG to IM to GAC. Increased trend of Lactobacillus and Lachnospiraceae with carcinogenesis progression
Eun et al., 2014 [33]	GAC (11) IM (11) NAG (10)	16S rRNA sequencing	In GAC group, Family Helicobacteraceae decreased significantly, whereas Bacilli and Streptococcaceae increased
Zhang et al., 2015 [34]	HP+ (8) HP− (14)	Whole genome sequencing	Increased *Staphylococcus epidermidis* but decreased *H. influenza* and *H. parainfluenza* among HP+
Jo et al., 2016 [35]	Healthy: HP+ (16) and HP− (13) GAC: HP+ (15) and HP− (19)	16S rRNA sequencing	Higher composition of Streptococcus, Stenotrophomonas, Ralstoni and Prevotellain the body mucosa of HP− GAC group.
Wang et al., 2016 [36]	NAG (212) GAC (103)	315 patients with quantitative PCR; 12 patients (6 with GC) received 16SrRNA sequencing	5 genera of bacteria with potential cancer-promoting activities (Lactobacillus, Escherichia-Shigella, Nitrospirae, *Burkholderia fungorum*, and Lachnospiraceae) were abundant in GC patients, of which Nitrospirae was found in all GC patients but was absent in all NAG
Tseng et al., 2016 [37]	GAC (6)	16S rRNA sequencing	Top genera before tumor resection: Ralstonia, Helicobacter, Lactobacillus, Stenotrophomonas, Burkholderia, Bacillus, Curvibacter, Bdellovibrio, Sulfuritalea and Legionella.
Li et al., 2017 [38]	NAG (9HP+) IM (9) GAC (7, tumors and non-tumors) HP−(controls)	16S rRNA sequencing	HP reduces bacterial diversity in HP−infected patients and its eradication restores microbial composition. GAC samples have reduced bacterial diversity. Top genera in HP−individuals: Haemophilus, Serratia, Neisseria and Stenotrophomonas
Yu et al., 2017 [39]	160 GAC patients (80 cardia GAC from China and 80 non-cardia GAC from Mexico)	16S rRNA sequencing	Top genera in non-malignant tissue: Helicobacter, Enterobacteriaceae (Chinese subgroup), and Streptococcus and Lactobacillus (Mexican subgroup).
Coker et al., 2018 [40]	Superficial gastritis (21) AG (23) IM (17) GAC (20, tumor and non-tumor)	16S rRNA sequencing	Higher abundance and strong co-occurrence of oral bacteria in GAC. Top genera enriched in GAC: Streptococcus, Lactobacillus, Peptostreptococcus, Gemella and Fusobacterium
Schulz et al., 2018 [41]	HP+ (8)	16S rRNA sequencing	Significant difference only in the relative abundance of Proteobacteria in HP patients, due to HP dominance.
Ferreira et al., 2018 [42]	GAC (54) Chronic gastritis (81)	16S rRNA sequencing	Patients with GAC had an over-expression of Actinobacteria, Firmicutes and non-HP Proteobacteria. Citrobacter, Clostridium, Lactobacillus, Achromobacter, and Rhodococcus were significantly more abundant in GAC patients.
Hsieh et al., 2018 [43]	Gastritis (9) IM (7) GAC (11)	16S rRNA sequencing	Patients with GAC had an abundance of Clostridium, Fusobacterium, and Lactobacillus.
Hu et al., 2018 [44]	Superficial gastritis (5) GAC (6)	Shotgun metagenomics	Differences in composition and function of the microbiota between superficial gastritis and GAC. Increased relative abundance of oral pro-inflammatory bacteria in GAC: genera Neisseria, Alloprevotella and Aggregatibacter, and species Streptococcus_mitis_oralis_pneumoniae and strain Porphyromonas_endodontalis.t_GCF_000174815.
Liu et al., 2019 [45]	GAC (276)	16S rRNA sequencing	HP is decreased in the tumoral microhabitat and has a negative co-occurrence with Prevotella, Bacteroides, Faecalibacterium, Phascolarctobacterium and Roseburia. Streptococcus, Selenomonas, *Prevotella melaninogenica*, *Streptococcus anginosus* and Propionibacterium acnes were enriched in the tumoral microhabitat.

HP: Helicobacter pylori; HP+: HP−positive; HP−: HP−negative; GAC: gastric adenocarcinoma; IM: intestinal metaplasia; AG: atrophic gastritis; FD: functional dyspepsia; NAG: non-atrophic gastritis; qPCR: quantitative PCR; T-RFLP: terminal restriction fragment length polymorphism.

**Table 2:** Immune checkpoint inhibitors studied in the context of gastric cancer.

Immune Checkpoint	Immune Checkpoint Inhibitors	Trade Name (Manufacturer)	Study Design	Results	Reference
CTLA-4	Tremelimumab	(AstraZeneca)	Phase II study in the 2nd line treatment of metastatic gastric cancer	4 patients stable disease 1 patient partial response	Ralph et al., 2010 [74]
	Ipilimumab	Yervoy (Bristol-Myers-Squibb)	Phase II study of ipilimumab versus best supportive care (BSC) in patients with advanced gastric cancer	PFS with ipilimumab versus BSC was not improved	Bang et al., 2017 [75]
PD-1	Nivolumab	Opdivo (Bristol-Myers-Squibb)	Attraction-2: A randomised, double-blind, placebo-controlled, phase 3 trial of Nivolumab in heavily pretreated gastric cancer patients	Median overall survival significantly better in Nivolumab group versus placebo	Kang et al., 2017 [76]
	Pembrolizumab	Keytruda (Merck Sharpe & Dohme corp.)	Keynote-059: A phase II trial of perbrolizumab monotherapy in previously treated gastric cancer patients Keynote-061: Phase III trial of pembrolizumab versus paclitaxel as 2nd line therapy	11.6% had objective response rate and 2.3% had complete response Pembrolizumab did not significantly improve overall survival compared with paclitaxel	Fuchs et al., 2018 [77] Shitara et al., 2018 [78]
	Toripalimab	(Shanghai Junshi Bioscience Co.)	Phase Ib/II trial evaluating the safety and activity of toripalimab in chemo-refractory (cohort 1) and chemo-naïve (cohort 2) gastric cancer patients	Cohort 1: ORR 12.1%, disease control rate (DCR) 39.7%. Cohort 2: ORR was 66.7% and the DCR was 88.9%	Wang et al., 2019 [79]
PD-L1	Avelumab	Bavencio (EMD Serono)	the JAVELIN Solid Tumor JPN trial: Phase 1 evaluating Avelumab in stage IV gastric cancer patients receiving prior therapy	objective response rate was 10.0% and median overall survival was 9.1 months	Doi et al., 2019 [80]
	Durvalumab	Imfinzi (AstraZeneca)	Phase Ib/II study in patients (pts) with metastatic or recurrent gastric cancer: D arm: received Durvalumab. T arm: received Tremelimumab and D+T arm: received Durbalumab and Tremelimumab	D+T has a manageable safety profile in 2L and 3L advanced gastric cancer, with encouraging OS versus D monotherapy	Kelly et al., 2018 [81]

## Abbreviations

TLRs	Toll-like receptors
NLRs	Nod-like receptors
PPR	Pattern recognition receptor
MAMPs	Micro-organism-associated molecular patterns
PAMPs	Pathogen-associated molecular patterns
MyD88	Myeloid differentiation response-88
TRIF	TIR-domain-containing, adapter-inducing interferon-β
NFkB	Nuclear factor kB
IRF	Interferon regulatory factor
AP-1	Activator protein-1
TNF-α	Tumor necrosis factor-α
Cdx2	Caudal type Homebox 2
IP-10	Interferon gamma-induced protein 10
IL	Interleukin
ROS	Reactive oxygen species
H2S	Hydrogen sulfide
H. pylori	Helicobacter pylori
MALT	Mucosa-associated lymphoid tissue
CagA	Cytotoxin-associated gene A
VacA	Vacuolating cytotoxin A
OLGA	Operative link on gastritis assessment
GAC	Gastric adenocarcinoma
IM	Intestinal metaplasia
AG	Atrophic gastritis
FD	Functional dyspepsia
NAG	Non-atrophic gastritis
T-RFLP	Terminal restriction fragment length polymorphism
LAB	Lactic acid bacteria
MCT-1	Monocarboxylate transporter 1
HIF-1	Hypoxia-inducible factor-1
HCAR1	Hydrocarboxylic acid receptor 1
MHC	Major histocompatibility complex
TCR	T-cell receptors
PD-1	Programmed cell death protein-1
LAG 3	Lymphocyte-activation gene 3
TIM 3	T cell immunoglobulin and mucin domain containing-3
TIL	Tumor infiltrating lymphocytes
IFN γ	Interferon γ
Tregs	T regulatory cells
CTLA-4	Cytotoxic T lymphocyte–associated protein 4
HMGB1	High mobility group box 1
NSCLC	Non-small cell lung cancer
CPS	Combined positive score
TPS	Tumor proportion score
MSI	Microsatellite instability
dMMR	Deficient MisMatch Repair
EBV	Epstein-Barr Virus
TMB	Tumor mutational burden
FMT	Fecal microbiota transplantation
